# Hypertonic solution-induced preconditioning reduces inflammation and mortality rate

**DOI:** 10.1186/s12950-019-0220-4

**Published:** 2019-07-03

**Authors:** Rosangela Nascimento Pimentel, Ricardo Costa Petroni, Hermes Vieira Barbeiro, Denise Frediani Barbeiro, Mariana Macedo Andrade, Suely Kumini Ariga, Francisco Garcia Soriano

**Affiliations:** 1 Laboratório de Investigação Médica – LIM 51, Faculdade de Medicina, Universidade de São Paulo (FMUSP), São Paulo, Brazil; 2 Emergências Clínicas do Departamento de Clínica Médica da Faculdade de Medicina da Universidade de São Paulo, Av Dr Arnaldo 455, room 3189, São Paulo, CEP 01246-903 Brazil

**Keywords:** Sepsis, Inflammation, Preconditioning, Hypertonic solution, Tolerance

## Abstract

**Background:**

Dysregulated inflammatory response is common cause of organ damage in critical care patients. Preconditioning/tolerance is a strategy to prevent exacerbated inflammation. The aim of this study is to analyze hypertonic saline 7.5% as a potential inducer of preconditioning that protect from a lethal dose of LPS and modulates systemic inflammatory profile in mice.

**Methods:**

Male Balb/C mice received intravenous (i.v.) injections of Hypertonic solution (NaCl 7.5%) (0.8 ml) for 3 days, on day 8th was challenged with LPS 15 mg/kg. Controls with Saline 0.9%, urea and sorbitol were performed. Microarray of mRNA expression was analyzed from HS versus saline from macrophages to identified the pathways activated by HS.

**Results:**

HS preconditioning reduced mortality after LPS injection as well reduced the cytokines release in plasma of the animals challenged by LPS. In order to check how HS induces a preconditioning state we measured plasma cytokines after each HS infusion. Repeated HS injections induced a state of preconditioning that reprograms the inflammatory response, resulting in reduced inflammatory cytokine production. A microarray of mRNA demonstrated that Hypertonic solution increased the expression of several genes in special Mapkbp1 and Atf3.

**Conclusion:**

hypertonic solution induces preconditioning/tolerance reducing mortality and inflammatory response after LPS challenge.

## Introduction

Ischemia/reperfusion (IR), systemic inflammatory response (SIRS) and sepsis present dysregulated inflammatory response in different diseases, and are the main causes of hospitalization in the US and worldwide [1, 2]. In addition to this, SIRS, IR and sepsis frequently affect surgical patients. These patients’ common cause of death is shock which is non responsive to vascular contractile drugs. Preconditioning is used in some surgical situations in order to protect patients from vascular clamp and consequent ischemia [3–7]. Several studies have shown the therapeutic potential of hypertonic solutions as treatment of different insults (sepsis, IR, SIRS). Studies with hypertonic sodium chloride solution revealed a prompt blood pressure restoration in experimental severe hemorrhagic shock [8–12]. Hypertonic solution treatment was effective in reducing mortality rate of endotoxemic rats [13], as well as prevented lung injury by LPS in the experimental model of ARDS [14, 15]. Hypertonic solution has been used in sepsis’ experimental studies and in small clinical studies [9, 16, 17]. In these studies, hypertonic solution improved tissue perfusion and reduced inflammation. On the other hand, hyperosmolarity can be a side effect of hypertonic solution used for therapeutic purposes.

Recent studies showed that hyperosmolarity causes cell stress activating intracellular MAP kinases pathway [18–20]. As a consequence of osmotic stress there is an activation of a nonspecific inflammatory response with release of cytokines [20]. IL1β in human aortic endothelial cells, IL6 in peritoneal macrophages of rats and IL8 in human peripheral blood mononuclear cells [9, 17, 21]. Small stress with different substances (e.g. LPS or cytokines) repeated several times can induce a protection [22–24]. Taking in account the fact that osmotic stress induces an inflammatory response through MAPK pathway we hypothesized that small doses of hypertonic solution injected days before a LPS challenge could induce also a tolerance or preconditioning state in mice. The advantage of hypertonic solution is lower toxicity as inducer of tolerance in patients compared to LPS or cytokines. Thus, this strategy can be used in surgical patients at high risk to develop SIRS, ischemia/reperfusion or sepsis.

The aim of this study was to analyze hypertonic saline 7.5% as a potential inducer of preconditioning in mice, protecting animals from a lethal dose of LPS and modulating the systemic inflammatory profile when administrated previously to LPS.

## Material and methods

### Mice

Adult male Balb/c mice (8 weeks old, weighing 20–25 g) were used for experiments. The animals provided from the School Facility were specific pathogen-free (SPF). Animals were maintained in a climate-controlled facility with an automatic light/dark cycle, with food and water available ad libitum. All procedures were performed in accordance with the Guide for the Care and Use of Laboratory Animals published by the US National Institutes of Health. The study protocol was approved by the Research Ethics Committee of the São Paulo School of Medicine (#0525/13).

### Preconditioning test – doses of hypertonic saline (NaCl 7.5%)

Mice were divided into three groups. 1- Group 2 ml/kg: animals received a dose of 2 ml/kg of NaCl 7.5% by intravenous tail injection at day one. The same dose was given at day three and day five. 2- Group 4 ml/kg: animals received a dose of 4 ml/kg of NaCl 7.5% by intravenous tail injection at day one. The same dose was given at day three and day five. 3- Group 8 ml/kg: animals received a dose of 4 ml/kg of NaCl 7.5% by intravenous tail injection at day one, a dose of 6 ml/kg of NaCl 7.5% by intravenous tail injection at day three and a dose of 8 ml/kg of NaCl 7.5% by intravenous tail injection at day five. At day 8 animals received an intraperitoneal injection of 10 mg/kg of LPS.

### Preconditioning test – uses of different solutions at similar osmolarity of HS solution - 2450 mOsm

In order to verify whether preconditioning effect of HS depends only of the solute NaCl or other solutes can induce a similar preconditioning state, we injected Urea and Sorbitol solutions at 45% concentration to match 7.5% NaCl osmolarity. Mice were divided into three groups. 1- Group HS: animals received a dose of 4 ml/kg of 7.5% NaCl by intravenous tail injection at day one, a dose of 6 ml/kg of 7.5% NaCl by intravenous tail injection at day three and a dose of 8 ml/kg of 7.5% NaCl by intravenous tail injection at day five. 2- Group HU animals received a dose of 4 ml/kg of 45% Urea by intravenous tail injection at day one, a dose of 6 ml/kg of 45% Urea by intravenous tail injection at day three and a dose of 8 ml/kg of 45% Urea by intravenous tail injection at day five. 3- Group HB animals received a dose of 4 ml/kg of 45% Sorbitol by intravenous tail injection at day one, a dose of 6 ml/kg of 45% Sorbitol by intravenous tail injection at day three and a dose of 8 ml/kg of 45% Sorbitol by intravenous tail injection at day five. At day 8 animals received an intraperitoneal injection of 10 mg/kg of LPS.

Negative controls for hypertonic solution were checked. NaCL at 0.9 and 3.5% NaCl, 20% Manitol were used at the same volume and injections time for check survival after LPS challenge.

### Survival rate

After preconditioning, the animals were injected with lethal dose of LPS (15 mg/Kg; i.p.) monitored three times daily. We analyzed the survival rate for 15 days (360 h); after which, moribund mice were euthanized by CO_2_ inhalation.

### Plasma

Blood samples were collected by means of cardiac puncture immediately before the mice were sacrificed. The samples were centrifuged at 1000 g (4 °C) for 10 min and the supernatant (plasma) was placed in Eppendorf plastic tubes and stored at − 80 °C for subsequent analysis.

### Cytokines

The concentrations of interleukin IL-α, IL-1β, IL-2, IL4, IL6, IL10, IL12 p40, IL12 p70, IL-17A, IL-17F, IL-22, IL-23, IFN-γ, macrophage inflammatory protein (MIP) -1 α, MIP-1 β and tumor necrosis factor α (TNF-α) in the plasma were determined by MilliPlex technology (# MCYTOMAG-70 K, Merck KGaA, Darmstadt, Germany). The samples were analyzed on a MagPix system, and the data were collected by Luminex xPONENT software.

### Macrophages

Peritoneal macrophages were collected from peritoneum cavity after saline or hypertonic infusion. Peritoneal cavity was washed with PBS buffer, the fluid collected was centrifuged and the pellet collected for RNA preparation.

### Preparation of RNA and microarray hybridization

Total RNA was extracted from frozen peritoneal macrophage using TRIZOL Reagent (Life technologies, Carlsbad, CA, USA). The integrity and quality of the RNA was assessed using the Bioanalyzer (Agilent Technologies, Santa Clara, CA, USA). RNA integrity number (RIN) values were ≥ 7.0. Isolated RNA was further purified using an RNeasy Mini Kit (Qiagen, Hilden, Germany). Purified total RNA was amplified by in vitro transcription and converted to sense-strand cDNA using a WT Expression kit (Ambion/Applied Biosystems, Foster City, CA, USA). cDNA was fragmented and labeled using a GeneChip WT Terminal Labeling kit (Affymetrix, Santa Clara, CA, USA). Fragmented cDNA samples were then hybridized to GeneChip Mouse Gene 1.0 ST Arrays (Affymetrix, Santa Clara, CA, USA). Images were processed and GeneChip Command Console Software (Affymetrix) were used to generate cell intensity files (CEL files). CEL files were imported into Expression Console and normalized using robust multiarray average (RMA).

### Gene Chip microarray analysis

Raw data from gene chips were summarized using RMA, which involves quantile normalization. Genes showing a statistically significance (*p* < 0.05) and a log2-transformed fold change of at least ±1.5 were identified as differentially expressed. Microarray data were validated using qRT-PCR for 8 selected DEGs, which demonstrated high correlation between microarray and qRT-PCR expression levels.

### Statistical analysis

All values are expressed as mean ± standard errors of the mean (SEM). Statistical analysis was performed using InStat Statistical Software (GraphPad, La Jolla, CA, USA). Comparisons between the experimental groups were made by analysis of variance (ANOVA) or Kruskal-Wallis. A Tukey test was used as a post hoc test to compare individual groups. A log-rank test was used to analyze survival. A *p* value less than 0.05 was considered to be significant.

## Results

**Fig. 1:**
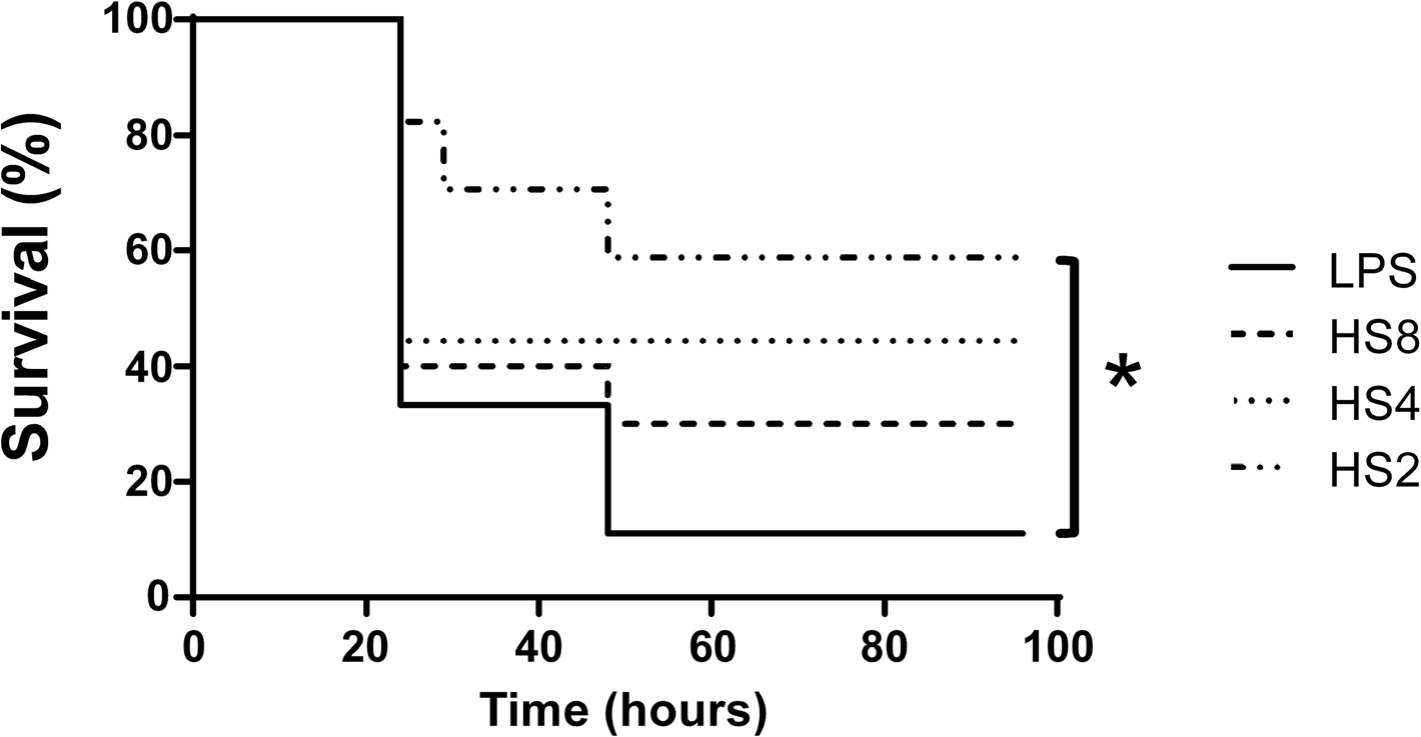
Effect of the preconditioning with different doses and concentrations of NaCl 7,5% in endotoxemic animals. **A-** Animals were separated in 4 groups – **LPS** (received a lethal dose of LPS 15 mg/kg i.p); **HS2** (received 3 doses in alternated days of 2 mL/kg of NaCl 7.5% and a lethal dose of 15 mg/kg of LPS at the 8th day of the study); **HS4** (received 3 doses of 4 mL/kg of NaCl 7.5% and a lethal dose of 15 mg/kg of LPS at the 8th day of the study); **HS8** (received 4 mL/kg at day one, 6 mL at day 3 and 8 mL/kg at day 5 and a lethal dose of 15 mg/kg of LPS at the 8th day of the study). *p < 0,05 vs LPS; were used 20 animals for each group. **B-** Animals were separated in 5 groups – **LPS** (received a lethal dose of LPS 15 mg/kg i.p); **HS7.5%** (received 4 mL/kg at day one, 6 mL at day 3 and 8 mL/kg at day 5 and a lethal dose of 15 mg/kg of LPS at the 8th day of the study); **HS3.5%** (received 4 mL/kg at day one, 6 mL at day 3 and 8 mL/kg at day 5 and a lethal dose of 15 mg/kg of LPS at the 8th day of the study); N**S0.9%** (received 4 mL/kg at day one, 6 mL at day 3 and 8 mL/kg at day 5 and a lethal dose of 15 mg/kg of LPS at the 8th day of the study); and **HM20%** received 20% mannitol (received 4 mL/kg at day one, 6 mL at day 3 and 8 mL/kg at day 5 and a lethal dose of 15 mg/kg of LPS at the 8th day of the study). *p < 0,05 vs LPS; were used 20 animals for each group

Three different doses of Hypertonic Saline 7,5% (HS) (4, 6 and 8 mL/kg) in alternated days were tested to determine which dose of HS was effective in inducing a preconditioning. In the HS8 group (we used increasing doses 4, 6 and 8 mL/kg to avoid hyperchloremia of animals). A lethal dose of LPS at the 8th day was injected i.p. for the survival study. We observed on Fig. 1a that hypertonic saline pre-conditioning increased animal survival after LPS injection when used increased doses of HS at the final concentration of 8 mL/Kg (*p* < 0.05). Figure 1b shows results for negative controls comparing 7.5, 3.5 and 0.9% NaCl solutions and also 20% Manitol, the data shows effective reduction in mortality only in 7.5% NaCl solution. Animals injected with PBS buffer instead LPS did not present any death.

**Fig. 2:**
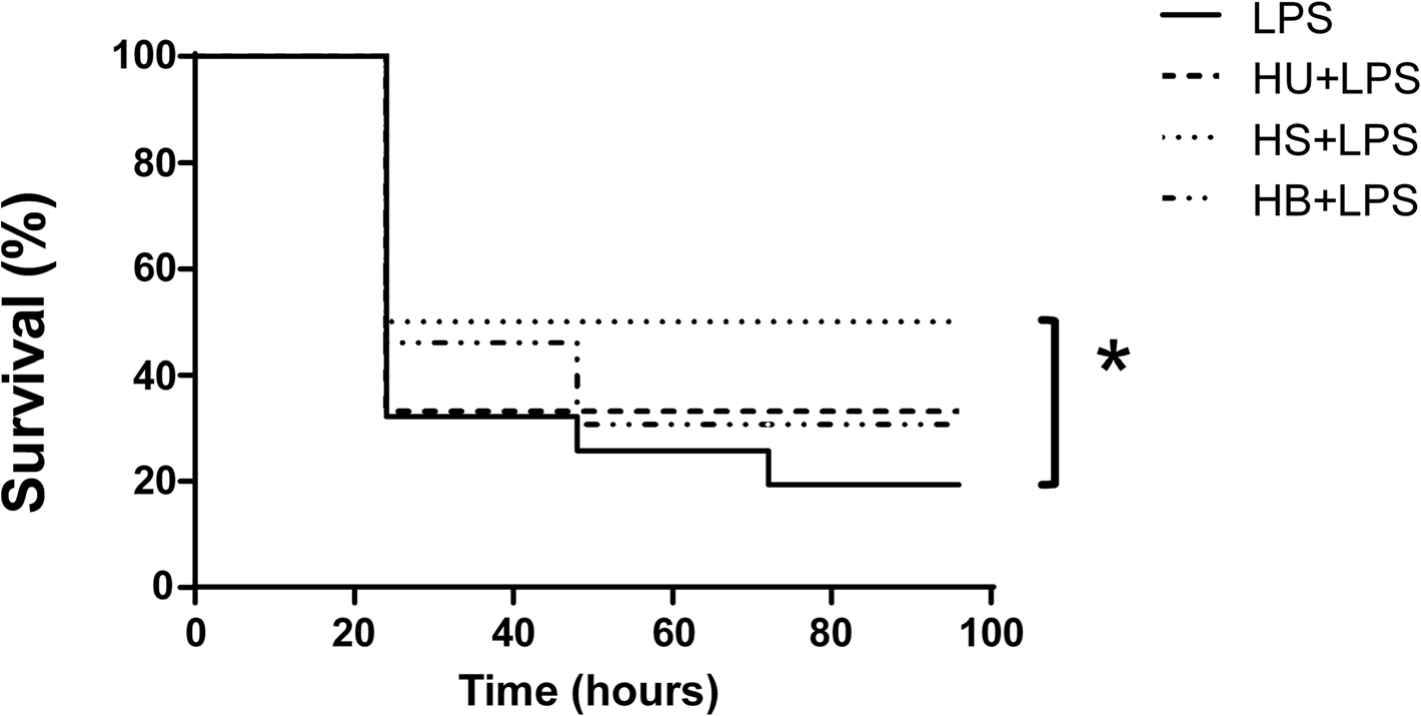
Effect of preconditioning with different solution before LPS injection. Balb/c mice were divided into groups: **LPS** (received a lethal dose of 15 mg/kg of LPS); HS + LPS (received 4 mL/kg on the first day, then 6 mL/kg on the third day and 8 mL/kg on the fifth day of Hypertonic Saline 7.5%, then on the eighth day a lethal dose of 15 mg/kg of LPS); HU + LPS (received 4 mL/kg on the first day, then 6 mL/kg on the third day and 8 mL/kg on the fifth day of Urea 45%, then on the eighth day a lethal dose of 15 mg/kg of LPS); HB + LPS (received 4 mL/kg on the first day, then 6 mL/kg on the third day and 8 mL/kg on the fifth day of Sorbitol 45%, then on the eighth day a lethal dose of 15 mg/kg of LPS). * *p* < 0,05 vs LPS group; were used 20 animals for each group

Figure 2 shows the results of different solute for hyperosmotic solution on mortality rate after a lethal dose of LPS. The protective effect was related to sorbitol, urea and HS with the same osmotic value. Taking in account our results, we observed that preconditioning effect was osmolarity-dependent. Therefore, we looked at different solutions with the same osmolarity to find out if the preconditioning effect observed by the hypertonic saline solution would be reproducible using other solutions. We observed that all solutions were able to induce a preconditioning state and increased survival rate compared to LPS group (*p* < 0.05).

**Fig. 3:**
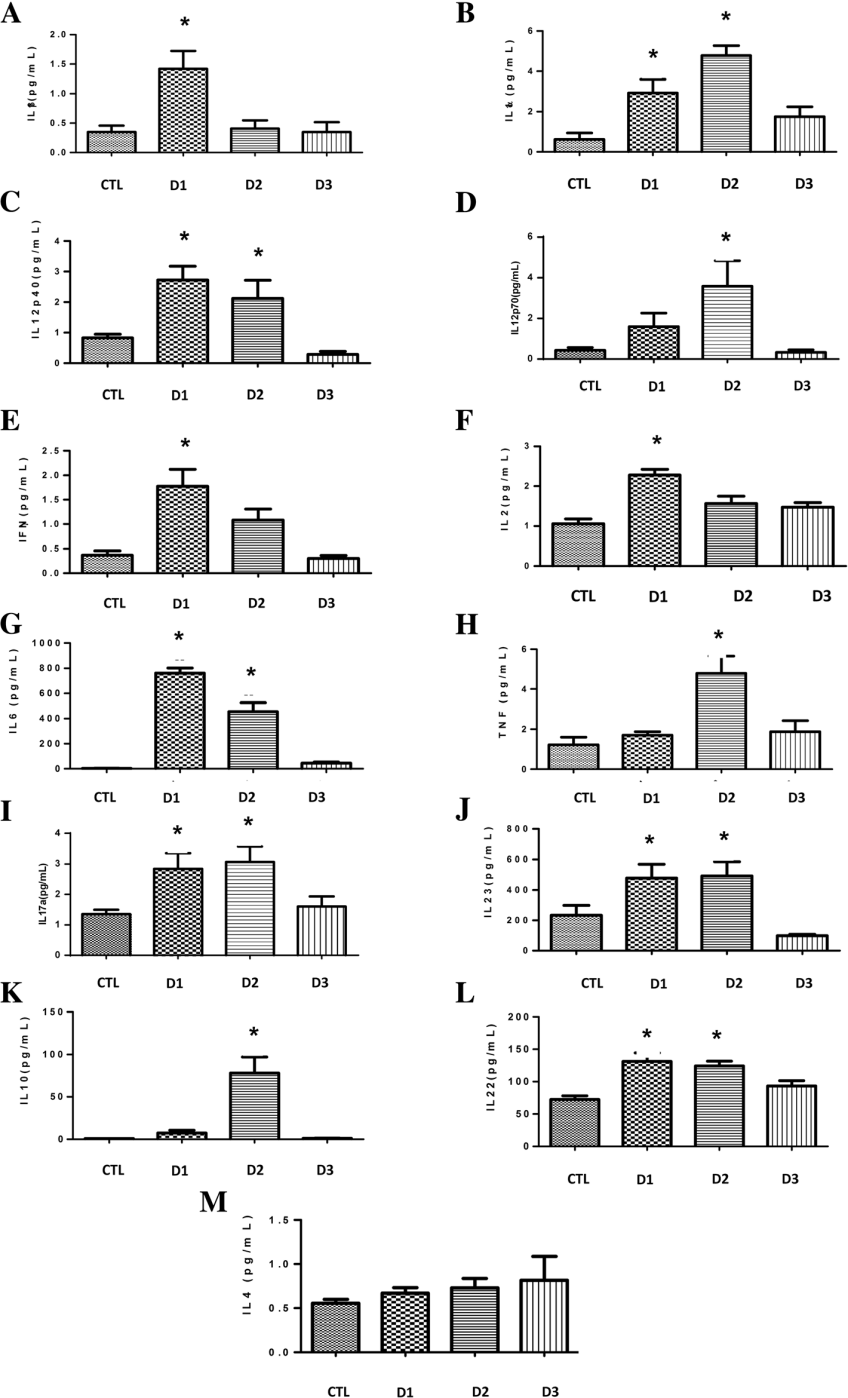
Effect of HS preconditioning on the cytokines concentrations. Pro inflammatory (**a**-**j**) and Anti-inflammatory (**k**-**m**) cytokines were analyzed. Balb/c mouse serum was collected after 4 h. Animals were allocated in 4 groups: CTL (Without treatment or insult); D1 (received one dose of 4 mL/kg of HS 7.5%); D2 (received one dose of 4 mg/kg of HS 7.5% at day one and one dose of 6 mL/kg of HS 7.5% at day three); D3 (received one dose of 4 mL/kg of HS 7.5% at day one, one dose of 6 mg/kg of HS 7.5% at day three and one dose of 8 mL/kg of HS 7.5% at day five). The data are represented as the mean ± SD, *n* = 8 animals. * *p* < 0.05 vs other groups, and # *p* < 0.05 vs CTL

Preconditioning process was analyzed by measuring different plasmatic cytokines production. The cytokines peak presents two different patterns: Cytokines that increased their expression after first dose and then maintaining elevated expression until second dose, decreasing after third dose (Fig. 3a, c, e, f, g, i, j, l) and the cytokines that increased their expression after a second dose and decreased their expression after third dose (Fig. 3b, d, h, k).

In order to identify the possible pathway used by the hypertonic solution to induce preconditioning, we performed a micro array assay in the peritoneal macrophages of animals. Gene expression array showed an important change in several genes exposed to hypertonic solution (Table 1). For example, gene related to cytokines receptors were reduced after hypertonic solution i.e. il1r2, and Mmp8. On the other hand, hypertonic solution increased Mapkbp1 and Atf3 that is related to hypertonic activation of cell signaling to preconditioning.

**Table 1:** Gene expression comparing hypertonic solution infusion vs. 0.9% NaCl in macrophages collected from peritoneal cavity

Gene	*p*-value	Fold Change
		Reduced
Lilrb4	0.0005	− 195.9
Slc7a11	0.0001	− 193.9
Il1r2	0.0005	− 190.9
Mmp8	0.0001	− 180.5
Scfd1	0.0001	− 179.9
Mgam	0.0001	− 162.1
Ptgs2	0.0005	− 157.0
		Increased
Mapkbp1	0.0001	166.9
Atf3	0.0001	150.3
Ptprg	0.0005	150.8
Gm94	0.0005	15.5
Ankrd	0.0002	16.5
Tmem126a	0.0005	1.6

**Fig. 4:**
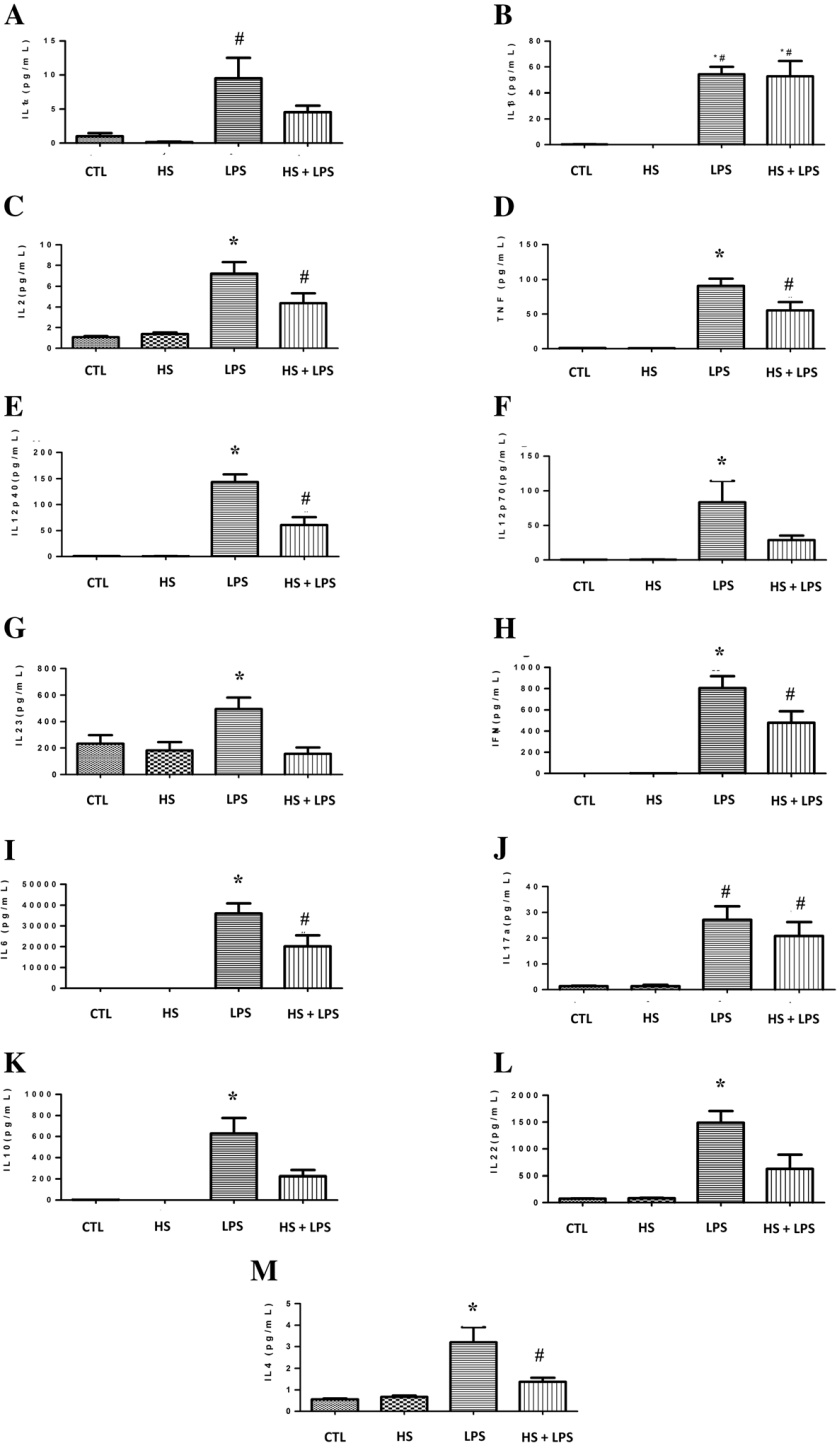
Effect of HS preconditioning on the modulation of systemic inflammation after LPS injection. Pro inflammatory (**a**-**j**) and Anti-inflammatory (**k**-**m**) cytokines were analyzed. Balb/c mouse serum was collected 4 h after LPS injection (i.p.). Animals were allocated in 4 groups: CTL (received no injury or treatment); HS (received one dose of 4 mg/kg of NaCl 7.5% at day one, one dose of 6 mg/kg of NaCl 7.5% at day three and one dose of 8 mg/kg at day five); LPS (received 15 mg/kg lipopolysaccharide i.p.); HS + LPS (received 4 mg/kg at day one, 6 mg at day 3 and 8 mg/kg at day 5 and a lethal dose of 15 mg/kg of LPS at the 8th day). The data are represented as the mean ± SD, n = 8 animals. * *p* < 0.05 vs other groups, # *p* < 0.05 vs CTL and HS groups

**Fig. 5:**
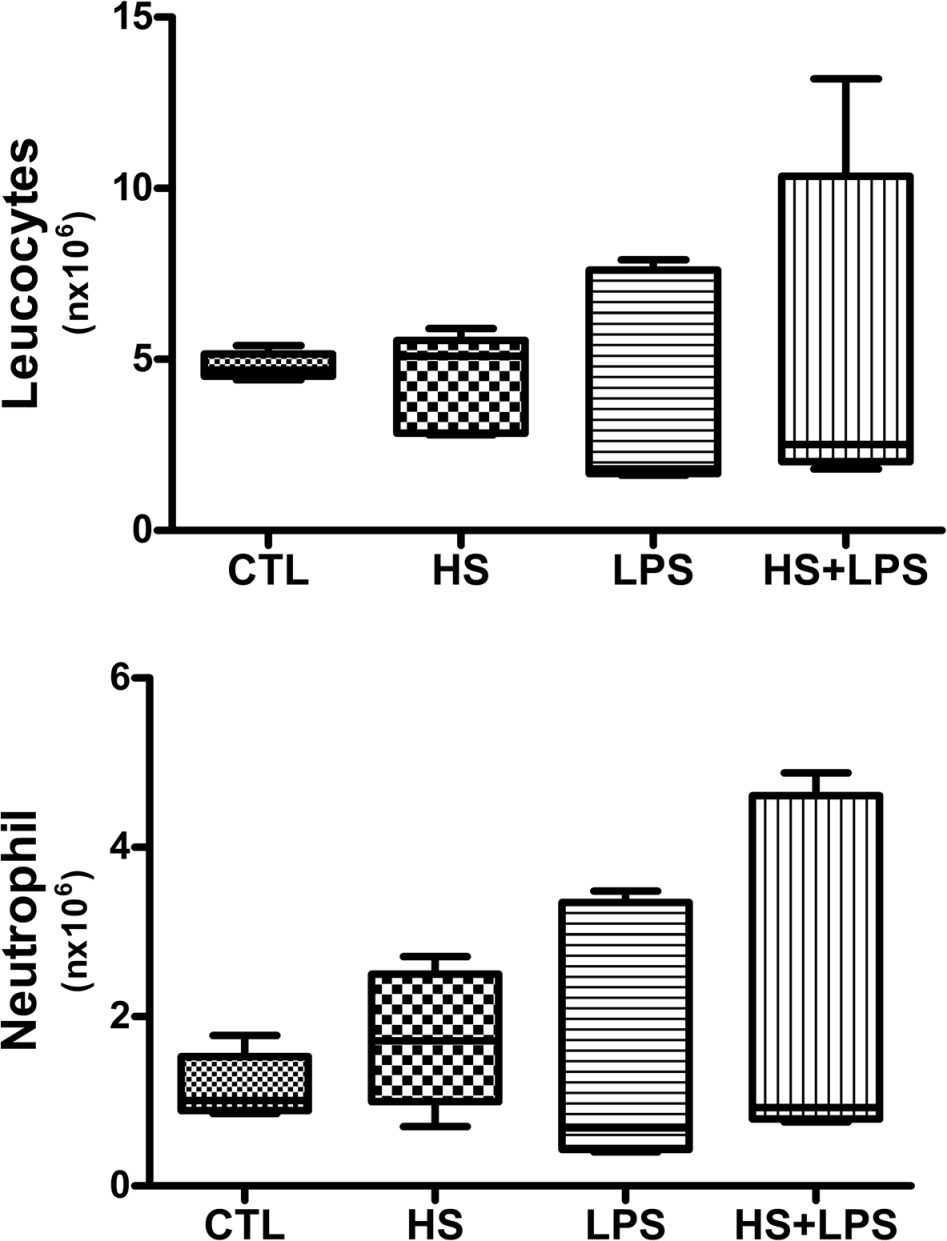
Effect of HS preconditioning on the leukocytes and neutrophil after LPS injection. Leukocytes (**a**) and neutrophils (**b**) were analyzed. Balb/c mouse blood collected 4 h after LPS injection (i.p.). Animals were allocated in 4 groups: CTL (received no injury or treatment); HS (received one dose of 4 mg/kg of NaCl 7.5% at day one, one dose of 6 mg/kg of NaCl 7.5% at day three and one dose of 8 mg/kg at day five); LPS (received 15 mg/kg lipopolysaccharide i.p.); HS + LPS (received 4 mg/kg at day one, 6 mg at day 3 and 8 mg/kg at day 5 and a lethal dose of 15 mg/kg of LPS at the 8th day). The data are represented as the median ± CI, SD, *n* = 6 animals

After confirming the potential effect of HS preconditioning in regulating the immune response, we studied whether HS preconditioning was capable to modulate systemic inflammatory response of animals subjected to endotoxemia by intraperitoneal LPS injection (Fig. 4). As expected, HS preconditioning decreased all cytokines production analyzed in animals treated before endotoxemia induction compared to animals without treatment (*p* < 0.05) (Fig. 4a, c, e, f, g, i, j, l, m). The effect of reducing cytokines production was not related to a decrease of leucocytes or neutrophils in the blood of animals which were induced to preconditioning as shown in Fig. 5. Thus, in accordance of our hypothesis, HS preconditioning avoided systemic inflammation of endotoxemic animals.

## Discussion

Although the benefits of HS treatment in modulate different injuries are stablished in literature, there are no studies showing the potential of HS in vivo developing preconditioning state able to modulate inflammation. Our study provides, for the first time, data showing that repeated IV infusion of Hypertonic Solution (HS) induces a preconditioning state able to protect animals from lethal LPS injection. We showed that HS preconditioning reduced mortality rate by lethal LPS injection as well as reduced the cytokines release in plasma. Serum cytokine time course after each HS dose showed progressive preconditioning development. The mechanistic pathway related to this process involves the MAPK pathway as observed by our micro array results that showed an increasing in Mapkbp and Atf3 expression. It is noteworthy that MAPK is a kinase activated by osmotic stress and phosphorylated ATF3 which inhibit at the gene promoter the production of cytokines [25].

Experimental studies have shown some benefits of hypertonic solution compared to isotonic fluid for resuscitation after trauma and hemorrhagic shock [8, 21]. Hypertonic solutions (HS) have been shown to reduce systemic inflammation and organ failure associated with resuscitated hemorrhagic shock [21]. Here we showed that the pretreatment with increased doses of HS (4, 6 and 8 mL/Kg) was able to induce a preconditioning state in animals that significantly reduced mortality rate of endotoxemic animals. There is one study in vivo using the term preconditioning to HS previous to ischemia/reperfusion insult in mice [26]. However, in this study the HS was infused 1 h before ischemia/reperfusion in the animals, it is worth noting that so close infusion of HS can protect the animals not by a preconditioning effect but due to volume expansion. Other authors have verified in a similar model of ischemia/ reperfusion that HS infusion has more potent effect in different time points [27]. The authors reported that several parameters demonstrate that HS administered pre-reperfusion is more effective than administered pre-ischemia [27]. Preconditioning should present protection at any moment of infusion [21, 28].

The next question in our study was to verify whether preconditioning state was due to osmotic stress or to solute. The question is if this effect is specific to hypertonic saline or any other osmotic stress could result in similar changes [29]. Our results showed that every hyperosmolar solution were able to decrease mortality rate on endotoxemic animals, so preconditioning is dependent on hyperosmotic stress. Negative controls with lower osmotic solutions in special 0.9% NaCl did not show protection as HS. Other studies on macrophage culture report that the effect of hypertonic preconditioning with either NaCl or mannitol is only transient, returning macrophage function within 20 h after hypertonic preconditioning [29]. Our results confirm that preconditioning induce by HS was effective even after an interval of 48 h, in fact the protection last 10 days, that was the complete time of this study.

There are studies reporting that osmotic stress deflagrates intracellular signaling through mitogen-activated protein kinase (MAPK). The components of the MAPK cascades, TAK1 and MEKKs, activate IKK leading to NF-κB activation [30, 31]. In our analysis of gene expression comparing normal saline (NaCl 0.9%) versus hypertonic saline (7.5%) treatment we found several genes that were changed by hypertonic solution. In particular, we highlight the finding that Mapkbp1 increased 166-fold and Atf3 increased 150-fold in animals treated with hypertonic versus saline solution. The literature showed that hormones, nutrient depletion, osmotic shock, oxidative stress, DNA damage activate Mitogen-activated/stress protein kinase (MAPK/SAPK) pathways [25, 32]. MAPK induced the phosphorylation of transcription factors of the ATF/CREB family and regulated the transcription of target genes. Therefore, ATF3 negatively regulate transcription of pro-inflammatory cytokines. Genes that possess ATF/CREB promoter binding sites within close proximity of NF-κB sites, are ATF3 modulate NF-κB related transcription [25, 32]. Since HS induced preconditioning in animals, we hypothesized whether this preconditioning state would be able to protect animals from an acute inflammation developed by LPS injection. Our results showed that animals submitted to HS preconditioning had a decreased cytokines expression compared to animals without treatment, suggesting a therapeutically effect of HS preconditioning in controlling inflammation.

Finally, our study demonstrated for the first time that HS can induce preconditioning in vivo lasting at least 10 days. Preconditioning effect was dependent on osmotic stress, as several hyperosmotic solutions reproduces the protection to lethal LPS. HS cause alterations in inflammatory cells by modulation of different genes involved in osmotic stress (e.g. MAPK) observed by gene expression analysis. The possible mechanistic pathway is the activation of MAPK and ATF3 activation of cascade with final reduction in the production of cytokines.

## Conclusion

Preconditioning effect was dependent on osmotic stress, as several hyperosmotic solutions reproduces the protection to lethal LPS. HS cause alterations in inflammatory cells by modulation of different genes involved in osmotic stress (e.g. MAPK) observed by gene expression analysis. Osmotic preconditioning was a protective effect in vivo useful in clinical practice for the *pre-treatment of surgical patients* submitted to risk procedures such as: reperfusion after ischemia; systemic inflammatory response or surgeries with bacterial contamination.

## Data Availability

The data are available.

## Abbreviations

ANOVA: Analysis of variance
ARDS: Acute respiratory distress syndrome
Atf3: Activating Transcription Factor 3 gene
cDNA: deoxyribonucleic acid copy
CREB: cAMP-response element binding protein
DEGs: Differentially expressed genes
HS: Hypertonic solution
i.p: intra peritoneal
i.v: intra venous
IFN-γ: interferon
IKK: IκB kinase
IL (1β, 6, 8, etc.): interleukin
IR: Ischemia reperfusion
Kg: Kilogram
LPS: Lipopolysaccharide
MAP kinases: Mitogen Activated Protein Kinases
Mapkbp1: Mitogen-Activated Protein Kinase Binding Protein 1 gene
MEKKs: Mitogen-activated protein kinase extracellular signal-regulated kinase kinase kinases
Mg: Milligram
min: minutes
MIP: Macrophage inflammatory protein
Ml: Milliliter
Mmp8: metalloproteinase gene
mRNA: messenger ribonucleic acid
NF-κB: nuclear factor kappa B
PBS: phosphate buffer solution
qRT-PCR: Reverse transcription polymerase chain reaction quantitative real time
RIN: RNA integrity number
SEM: Standard errors of the mean
SIRS: Systemic Inflammatory Response Syndrome
SPF: Specific pathogen-free
TAK1: Transforming growth factor beta-activated kinase
TNF-α: tumor necrosis factor α
US: United States
